# Validity of self-reported substance use: research setting versus primary health care setting

**DOI:** 10.1186/s13011-021-00398-3

**Published:** 2021-09-14

**Authors:** Parvin Khalili, Ali Esmaeili Nadimi, Hamid Reza Baradaran, Leila Janani, Afarin Rahimi-Movaghar, Zohre Rajabi, Abdollah Rahmani, Zahra Hojati, Kazem Khalagi, Seyed Abbas Motevalian

**Affiliations:** 1 Department of Epidemiology, School of Public Health, Iran University of Medical Sciences, Tehran, Iran; 2 Social Determinants of Health Research Centre, Rafsanjan University of Medical Sciences, Rafsanjan, Iran; 3 Non-Communicable Diseases Research Center, Rafsanjan University of Medical Sciences, Rafsanjan, Iran; 4 Ageing Clinical & Experimental Research Team, Institute of Applied Health Sciences, University of Aberdeen, Aberdeen, Scotland, UK; 5 Preventive Medicine and Public Health Research Center, Psychosocial Health Research Institute (PHRI), Iran University of Medical Sciences, Tehran, Iran; 6 Department of Biostatistics, School of Public Health, Iran University of Medical Sciences, Tehran, Iran; 7 Iranian National Center for Addiction Studies (INCAS), Tehran University of Medical Sciences, Tehran, Iran; 8 Osteoporosis Research Center, Endocrinology and Metabolism Clinical Sciences Institute, Tehran University of Medical Sciences, Tehran, Iran; 9 Research Center for Addiction and Risky Behaviors (ReCARB), Psychosocial Health Research Institute (PHRI), Iran University of Medical Sciences, Tehran, Iran

**Keywords:** Substance use, Self-reported, Underreporting, Validity, Research setting, Primary health care settings

## Abstract

**Background:**

Self-reported substance use is more likely to be influenced by underreporting bias compared to the biological markers. Underreporting bias or validity of self-reported substance use depends on the study population and cannot be generalized to the entire population. This study aimed to compare the validity of self-reported substance use between research setting and primary health care setting from the same source population.

**Methods and materials:**

The population in this study included from Rafsanjan Youth Cohort Study (RYCS) and from primary care health centers. The sample from RYCS is made up 607 participants, 113 (18.62%) women and 494 (81.38%) men and sample from PHC centers is made up 522 individuals including 252 (48.28%) women and 270 (51.72%) men. We compared two groups in respect of prevalence estimates based on self-reported substance use and urine test. Then for evaluating validity of self-reported substance use in both group, the results of reference standard, urine tests, were compared with the results of self-reported drug use using measures of concordance.

**Results:**

The prevalence of substance use based on urine test was significantly higher in both settings compared to self-reported substance use over the past 72 h. The sensitivity of self-report substance use over the past 72 h in research setting was 39.4, 20, 10% and zero for opium, methadone, cannabis and amphetamine, respectively and in primary health care setting was 50, 20.7, 12.5% and zero for opium, methadone, cannabis and amphetamine, respectively. The level of agreement between self-reported substance use over the past 72 h and urine test indicated fair and moderate agreement for opium in both research and primary health care settings, respectively and also slight agreement for methadone and cannabis in both settings were reported. There was no significant difference between the two groups in terms of self-reported substance use. For all substances, the level of agreement increased with longer recall periods. The specificity of self-report for all substances in both groups was more than 99%.

**Conclusion:**

Individuals in primary health care setting were more likely to self-reported substance use than in research setting, but setting did not have a statistically significant effect in terms of self-reported substance use. Programs that rely on self-reported substance use may not estimate the exact prevalence of substance use in both research and primary health care settings, especially for substances that have a higher social stigma. Therefore, it is recommended that self-report and biological indicators be used for more accurate evaluation in substance use studies. It is also suggested that future epidemiological studies be performed to reduce bias of social desirability and find a method providing the highest level of privacy.

**Supplementary Information:**

The online version contains supplementary material available at 10.1186/s13011-021-00398-3.

## Background

Several epidemiologic studies have been performed to estimate the substance use in population-based samples of adults and adolescents worldwide including Iran [1, 2].

Accurate measurement of substance use among youth and adolescents are important to design and develop prevention programs. High-quality data can contribute to the efficient and appropriate use of limited resources and reduction in some high-risk behaviors. However, estimating the prevalence of substance use in a representative population may yet result in an inaccurate measurement due to underreporting [3]. Measurement error is considered as an observed difference between the real and the obtained values of a measurement [4]. Despite recent advances in biological assays which contributes to the improvement of measuring and evaluating the prevalence of substance use, researchers rely on self-reported information in the epidemiological studies to save time, cost, resources, and the possibility to collect required information on a larger scale [4, 5].

Substance use in societies, depending on cultural and social circumstances, is often considered as a sensitive, stigmatized, shameful and even illegal act, so self-reporting may be subject to secrecy, deception and bias [2, 5]. Therefore, for these reasons, the validity of the self-reported data has been questioned [6]. Numerous studies have shown that self-reported information about substance use cause underreporting in comparison with biological tests; that prone these studies susceptible to measurement errors such as self-reported bias and deviations of the real results [4, 6–11]. However, the amount of self-reported bias depends on the study population and cannot be generalized to the entire population [8]. Previous studies have shown that variation in the accuracy of self-reported data about substance use depending on substance type, age, data collection settings, education, socioeconomic status and region [12–14].

In Iran, the history of opium use as a recreational and medicinal substance dates back to more than four centuries [4]. Iran has the highest rate of opioid use in the world. In recent years, the use of heroin, ecstasy and crystal methamphetamine has increased. Based on the results of a national household survey, the prevalence of substance use in Iran, the most common of which was opium, estimated 2% [1]. However in Iran, due to some social and cultural beliefs and legal restrictions, it is not possible to estimate the absolute prevalence of illegal drugs [4, 15].

In recent years, one of source for gathering information on the prevalence of substance use in Iran were population-based epidemiological studies such as general population-based cohort studies. Rafsanjan Youth Cohort study (RYCS) as one the hub centers of Persian Youth Cohort (PYC) [16] was launched in 2016 in Rafsanjan city in order to evaluate wide range of psychiatric disorders and problems, such as illegal drugs and other high-risk behaviors, accidents, and injuries in a population of 15–34 years (born between 1982 and 2001) [17].

Thus, in RYCS, validity of information such as self-reported substance is questioned. Although, Abnet et al. reported a high rate of sensitivity of opium use in a population cohort study among Turkmen in northern of Iran [18],but it has been shown that the Turkmen population uses opium as a traditional medicine with a low social stigma [18]. Also, Ashrafi et al. showed that the validity of self-reported drug use was low in Azar population based cohort study [4].Thus, this sensitivity is not constant and may vary for different geographical areas in Iran as well as for different types of substances.

In addition, one other of source for gathering information on the prevalence of substance use and its severity among youth in Iran is the Integrated Health System (IHC) of the Ministry of Health. The Integrated Health System aimed to provide health services in the form of health system transformation plans and projects in Iran. All information on households, types of health services needed in community health centers and bases, and health homes is entered and recorded in this system. In other words, IHS has been designed and implemented with the aim of obtaining and updating information related to the health status of the Iranian population and maintaining this information in the population based national electronic health record (EHR) [19, 20]. The elementary version of the online system was presented to the Iran Ministry of Health in 2015 and has become to the source of collection epidemiological information from the community, for health policy makers as well as HCPs providing health care [21]. Thus, in PHC, validity of information such as self-reported substance is questioned.

Studies examined the self-report use of various drugs in comparison to biological tests have widely focused on specific samples in limited areas such as prisoners, patients, people in a particular occupation or people with substance use disorder, while population-based validation studies are scarce [4, 22]. Since the first step in the problem-solving process is always to determine the magnitude of the problem in terms of frequency, severity, and recognition of the current situation, accurate measurement of substance use are important to design and develop prevention programs. For these reasons, Determine and compare the validity of the self-reported substance use in research setting and primary health care setting, are important to design and develop prevention programs. However, to the best of our knowledge, little is known about the difference in the Validity of Self-Reported Substance Use in Research Setting and Primary Health Care Setting. For this reason, this study aimed to (i) examine and quantify whether prevalence of drug use in people who volunteered for Participation in youth cohort study differ from non-participants. (ii) Determining the concordance between results of self-reported drug use with the results of urine tests in the early phase of the Rafsanjan Youth Cohort Study. (iii) Determine and compare the validity of self-reported substance use between research setting and primary health care setting among youth and adolescents in Rafsnjan area in Iran, which selected from a geographical area with similar socioeconomic and cultural characteristics. The best of our knowledge, the current paper is the first study in this regard in the youth and adolescent population.

## Materials and methods

### Study sample

**Fig. 1:**
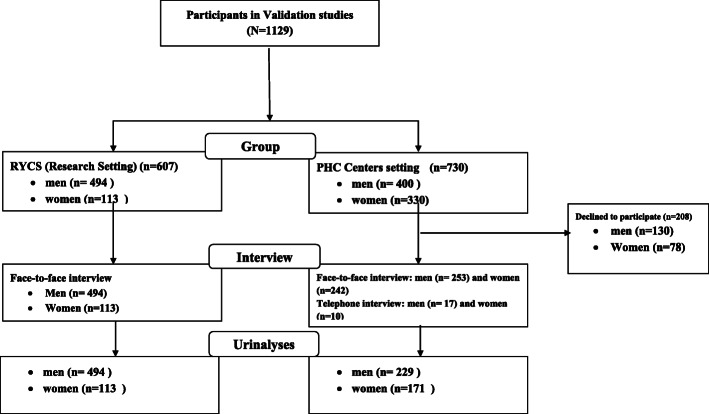
Flowchart of study sample, Rafsanjan Youth Cohort Study and PHC Centers setting

Rafsanjan Youth Cohort study (RYCS) is one of the academic centers of the PYC study carried out on 3000 youth population (aged 15–34 years/born in 1982–1991) which started in 2016 in both urban and rural areas of Rafsanjan city. A comprehensive questionnaire was asked by trained interviewers in the phase of baseline data collection and biological specimens (urine, blood, hair and nails) were collected from December 2016 to December 2018. Follow-up visits are in progress [17]. The current study was conducted on a subset of those invited to the RYCS including participants and non-participants (Fig. 1). The population in this study included from RYCS and from primary care health centers.

#### Rafsanjan youth cohort study (RYCS)

The sample from RYCS is made up 607 participants, 113 (18.62%) women and 494 (81.38%) men. All participants were included in the initial phase of the RYCS (baseline data collection) from March 2018 to December 2018.

#### Primary health care setting

Sample from PHC centers is made up 522 individuals including 252 (48.28%) women and 270 (51.72%) men. It should be noticed that participants from PHC centers were among those invited to participate in the RYCS at the initial phase but they were not willing to take part in the this cohort but they were recruited by PHC centers’ health officers for annual and periodic health screening tests and examination. We selected a subset of 730 individuals, including 330 women and 400 men, among those invited to participate in the RYCS but were not willing to take part in the study. Then, these people were asked to go to the primary health care center for a comprehensive assessment of their health status and a checkup by health care providers and physicians present at the health center (body mass index, lipid and blood glucose testing, oral and dental hygiene, mental, social and nutrition health). Participants, who participated through PHC, have no information about current research. Among these 253 male and 242 female referred to the health center to assess their health status and among them, 171 women and 229 men were willing to give blood and urine samples. Seventeen men and ten women only agreed to the telephone interview (Fig. 1).

#### Measures

##### Self-reported substance use

In RYCS, a large number of questionnaires were completed by well-trained psychologists, which one of is substance use Questionnaires.

In the RYCS group, participants were asked about any substance use over the past 12 months and 72 h. Questions were about opioid use including crude opium, syrup (extracts from opium residues) and burnt (opium residues), heroin and crack. Also, history of use of other substances including methadone, cannabis, amphetamine and methamphetamine was also questioned. For example, the questions regarding opium use (and other drug) were “Have you used opium over the past 12 months?”, If so, “Have you used opium over the past 72 hours?”

In the primary care health centers group, aforementioned which were part of the protocol of the Ministry of Health’s youth and middle ages program were asked by the health care providers at the health center when assessing health status. Health care providers were also public health or midwifery experts trained in the field.

All questionnaires were asked by trained interviewers. Four psychologists trained by a group of expert in order to interview with participants in the RYCS, in a one-day workshop. Also In the primary care health centers group, similar in the RYCS, Trained interviewers asked all questionnaires. For this purpose, five Health care providers trained by a group of expert in order to interview with participants in the primary care health centers, in a one-day workshop. All the questionnaires used in this study were prepared as a software program and the interviews were conducted face-to-face and individual responses were entered directly into the computer by interviewers. Experts introduced the study to the participants. If they were not willing to respond, they were excluded and the reasons for their refusal to study were recorded.

##### Urine drug screen

Approximately 5 ml urine sample was collected from all individuals were willing to give urine samples at the time of data collection. Urine samples were tested for morphine, methadone, cannabis, amphetamine and methamphetamine by using Urine Rapid Drug Screen (URDS).Thin Layer Chromatography (TLC) performed as the reference Standard to determine substance use. The TLC test was used for participants with a positive URDS test. In respect of initial screening, the URDS test was used to detect the existence of drug metabolites including morphine (cutoff point: 300 ng/ml), cannabis (cutoff point: 50 ng/ml), amphetamine and methamphetamine (cutoff point: 500 ng/ml), and methadone metabolites (cutoff point: 300 ng/ml). Metabolites of morphine and methadone can be detected in the urine by 72 h after being used and metabolites of amphetamine and methamphetamine can be detectable by 96 h after use [23]. Consequently, the TLC test was used for participants with a positive URDS test. The sensitivity of the URDS is above 98%. Sensitivities for the URDS compared to TLC ranged from 96% for cocaine to > 99% for Opioids [24]. Urine samples were analyzed by laboratory in Rafsanjan University of Medical Sciences.

#### Statistical analysis

To describe the data, mean and standard deviation for quantitative variables and frequency and percentage for qualitative variables were reported.

In this study, the results of standard tests (urine test) were compared with the results of self-report data about substance use by McNamara’s test. Conditional kappa, sensitivity and specificity were used for calculating agreement and concordance between laboratory tests and self-reported results in the past 72 h. Traditional Cohen’s kappa evaluates the agreement between the two criteria after correcting the effect of chance and accident, and considers the probability of equal error for both parties. In contrast, conditional kappa maintains just a variable such as urine test as criterion and after correcting the effect of chance, assesses the agreement of the other measurement according to the urine test [25]. The kappa values ≤0.20% considered as Poor, 0.21–0.40 Fair, 0.41–0.60 moderate, 0.61–0.80 Substantial, 0.81–1.00 Almost Perfect [26]. Moreover, Conditional kappa and sensitivity were also used to describe concordance between self-reported substance use in the past 12 months and laboratory test results. Specificity was not calculated for self-reported substance use over the past year, as some people may have correctly self reported substance use over the past year, but a urine test could not determine substance use over the past year, so it mistakenly could raise the number of false negatives. Finally, Chi square test and multivariable logistic regression model was used to assess the validity of self-reported substance use in during the past 72 h among urinalysis-confirmed users in group of Primary health care setting compared to group of RYCS. For sufficiently power analysis of low prevalence substances, in multivariable logistic regression model we collapsed all drugs. Variable gender include in the multivariable logistic regression model. All statistical analyses were performed through STATA version 14 except conditional kappa, which was calculated in Microsoft Excel.

#### Ethical considerations

The ethics committee of Rafsanjan and Iran University of Medical Sciences (#IR.RUMS.REC.1397.243 and IR.RUMS.REC.1398.042) approved this study. Written informed consent was obtained from the participants. The data of participants kept confidential, and urine samples were kept anonymous for researchers so that the results were not related to the identities of the participants in the dataset. To reach this goal, the urine samples were transported into the container with the unique number to identify whether each sample belonged to a male or female as well as the kind of substance being used.

## Results

This study included 1129 participants, 494 men (81.38%) and 113 women (18.62%) in the RYCS (Research Setting) group with the mean age of 28.04 ± 6.18 years and 270 men (51.72%) and 252 women (48.28%) in PHC setting group with the mean age of 29.11 ± 9.43 years. The details of demographic characteristics including age and gender was presented (Table 1).

**Table 1:** Demographic characteristics of the participants (*N* = 1129)

Variable		M (SD) or n (%)		p
		RYCS/ research setting	primary health care setting	
Sex	Male	494 (81.38)	270 (51.72)	–
	Female	113 (18.62%)	252(48.28)	
Age		28.04 ± 6.18	29.11 ± 9.43	0.023

### Prevalence of self-reported substance use in the RYCS (research setting) versus PHC (primary health care) setting

Table 2 presents the prevalence of each substance use estimated through urine test and self-reporting. Overall, regarding opium use, the prevalence of self-reported substance use in the past 72 h was statistically lower in men who were in the RYCS group (2.43%) than in the PHC group (6.99%) but not for other substance use, also the prevalence estimates based on urinalyses for all substance use were significantly higher in men of PHC group. In other words, men in PHC group, compared to men in RYCS group, were more often the recent substance user. Although, the prevalence of all substance use over the past 12 month was higher in men who were in the PHC group compared to the RYCS group, but there was no statistically significant difference between the two groups in this regard.

**Table 2:** Prevalence of self-reported and urinalysis-screened drug use at among participants (N = 1129)

				Self-reported drug use during the past 12 month			Self-reported drug use during the past 72 h			Positive urinalysis			Self-reported during the past 72 h versus urine test^a^
drug use	gender	group	N	n (%)	95%CI	p	n (%)	95%CI	P	n (%)	95%CI	p	p
**opioid use**	**Male**	**RYCS (Research Setting)**	**494**	61 (12.35%)	(9.72–15.57)	0.544	12 (2.43%)	(1.38–4.24)	0.003	32 (6.48)	(4.61–9.03)	0.003	< 0.001
		**PHC Centers setting** **(with urine specimen)**	**229**	32 (13.97%)	(10.03–19.14)		16 (6.99%)	(4.31–11.14)		30 (13.10)	(9.29–18.16)		< 0.001
		**PHC Centers setting** **(total)**	**270**	39(14.44)	(10.71–19.20)	0.412	19 (7.04)	(4.52–10.80)	0.002	–	–	–	–
	**Female**	**RYCS (Research Setting)**	**113**	6 (5.31%)	(2.38–11.44)	0.039	1 (0.88)	(0.12–6.18)	0.767	1 (0.88)	(0.12–6.18)	0.362	1.00
		**PHC Centers setting** **(with urine specimen)**	**171**	2 (1.17%)	(0.29–4.62)		1 (0.58)	(0.081–4.11)		4 (2.34)	(0.87–6.12)		0.250
		**PHC Centers setting** **(total)**	**252**	3 (1.19)	(0.38–3.04)	0.019	2 (0.79)	(0.20–3.15)	0.929	–	–	–	–
	**total**	**RYCS (Research Setting)**	**607**	67(11.04)	(8.77–13.80)	0.190	13(2.14)	(1.25–3. 66)	0.054	33(5.44)	(3.89–7.56)	0.056	< 0.001
		**PHC Centers setting** **(with urine specimen)**	**400**	34(8.50)	(6.13–11.68)		17 (4.25)	(2.65–6.74)		34 (8.50)	(6.13–11.68)		< 0.001
		**PHC Centers setting** **(total)**	**522**	42 (8.05)	(5.99–10.72)	0.090	21 (4.02)	(2.63–6.10)	0.065	–	–	–	–
**cannabis**	**Male**	**RYCS (Research Setting)**	**494**	8 (1.62%)	(0.81–3.21)	0.595	1 (0.20%)	(0.028–1.4)	0.192	10 (2.02)	(1.09–3.73)	0.001	0.004
		**PHC Centers setting** **(with urine specimen)**	**229**	5 (2.18%)	(0.71–5.02)		2 (0.87%)	(0.22–3.5)		16 (6.99)	(4.05–11.10)		< 0.001
		**PHC Centers setting** **(total)**	**270**	5 (1.85%)	(0.60–4.3)	0.812	2 (0.74)	(0.09–2.65)	0.255	–	–	–	–
**methadone**	**Male**	**RYCS (Research Setting)**	**494**	16 (3.24%)	(1.99–5.23)	0.302	4 (0.81%)	(0.30–2.14)	0.053	19 (3.85)	(2.46–5.96)	0.000	< 0.001
		**PHC Centers setting** **(with urine specimen)**	**229**	11 (4.80%)	(2.68–8.50)		6 (2.62%)	(1.17–5.74)		25 (10.92)	(7.46–15.70)		< 0.001
		**PHC Centers setting** **(total)**	**270**	12(4.44)	(2.53–7.69)	0.397	6(2.22)	(1–4.88)	0.101	–	–	–	–
	**female**	**RYCS (Research Setting)**	**113**	0 (0%)	0 (0%)	–	0 (0%)	0 (0%)	–	1 (0.88)		0.362	1.00
		**PHC Centers setting** **(with urine specimen)**	**171**	0 (0%)	0 (0%)		0 (0%)	0 (0%)		4 (2.34)			0.125
		**PHC Centers setting** **(total)**	**252**	0 (0%)	0 (0%)	–	0 (0%)	0 (0%)	–	–	–	–	–
	**total**	**RYCS (Research Setting)**	**607**	16 (2.64)	(1.51–4.25)	0.913	4 (0.66)	(0.18–1.68)	0.188	20(3.29)	(2.02–5.04)	0.004	< 0.001
		**PHC Centers setting** **(with urine specimen)**	**400**	11 (2.75)	(1.38–4.87)		6 (1.50)	(0.55–3.24)		29 (7.25)	(4.91–10.25)		< 0.001
		**PHC Centers setting** **(total)**	**522**	12 (2.30)	(1.19–3.98)	0.717	6 (1.15)	(0.42–2.48)	0.381	–	–	–	–
**amphetamine and Methamphetamine**	**Male**	**RYCS (Research Setting)**	**494**	0	0		0	0		1 (0.20)	(0.03–1.33)	0.557	1.00
		**PHC Centers setting** **(with urine specimen)**	**229**	0	0		0	0		2 (0.87)	(0.11–3.12)		0.50
		**PHC Centers setting** **(total)**	**270**	1	0.37	0.176	1	0.37	0.176	–	–	–	–

^a^ The results of standard tests (urine test) were compared with the results of Self-reporting of substance use during the past 72 h by McNamara’s test

In addition, the prevalence of opium use over the past 72 h was no significantly different between the two groups of women. The prevalence of opium use over the past 12 months was significantly different between the two groups of women. In other words, women in PHC group had lower self-report of opium use over the past 12 months compared to the RYCS group.

### Concordance between urine test and self-report substance use

As shown in table 2, based on McNamara’s test results, Regarding all drugs, the prevalence estimates based on urine test were significantly higher in both groups compared to self-reporting over the past 72 h (*p* < 0.001). Table 3 shows concordance value between self-report substance use with different recall periods and having a positive urine test. Sensitivity of self-report (i.e., number of self-reported substance use divided by detected substance users through a urine test) in men of RYCS group were 37.5, 21.1 and 10% for morphine, methadone and cannabis respectively and in the PHC group were 53.3, 24 and 12.5% over the past 72 h but with increasing the length of the recall period, this index increased in both groups.

**Table 3:** Determine and compare the validity of self-reported substance use in Rafsanjan Youth Cohort Study and primary health care setting (N = 1007)

	Rafsanjan Youth Cohort Study (research setting)						primary health care setting						compare the validity of self-reported substance use in two settings
Drug/gender	Sensitivity	95% CI	Specificity^a^	95% CI	Conditional κ	95% CI	Sensitivity	95% CI	Specificity^a^	95% CI	Conditional κ	95% CI	p-value^c^
opioid /total													
**past 72 h**	39.4	(22.9–57.9)	100	(99.4–100)	0.381	(0.376–0.385)	50	(32.4–67.6)	100	(99–100)	0.478	(0.470–0.486)	0.383
**past 12 months**	57.6	(39.2–74.5)	–	–	0.523	(0.522–0.524)	64.7	(46.5–80.3)	–	–	0.614	(0.608–0.620)	-^d^
opioid /men													
**past 72 h**	37.5	(21.1–56.3)	100	(99.2–100)	0.360	(0.353–0.366)	53.3	(34.3–71.7)	100	(98.2–100)	0.498	(0.482–0.515)	0.211
**past 12 months**	56.3	(37.7–73.6)	–	–	0.501	(0.499–0.503)	70	(50.6–85.3)	–	–	0.651	(0.640–0.660)	-^d^
opioid /women													
**past 72 h**	100	(2.5–100)	100	(96.8–100)	**1**	**1**	25	(.63–80.6)	100	(97.8–100)	0.246	(0.242–0.250)	0.171
**past 12 months**	100	(2.5–100)	–	–	**1**	**1**	25	(.63–80.6)	–	–			-^d^
Cannabis/men													
**past 72 h**	10	(.253–44.5)	100	(99.4–100)	0.10	(0.096–0.101)	12.5	(1.55–38.3)	100	(98.3–100)	0.117	(0.105–0.130)	0.846
**past 12 months**	20	(2.52–55.6)	–	–	0.187	(0185–0.189)	12.5	(1.55–38.3)	–	–	0.105	(0.094–0.117)	-^d^
Methadone/total													
**past 72 h**	20	(5.73–43.7)	100	(99.4–100)	0.195	(0.192–0.198)	20.7	(7.99–39.7)	100	(99–100)	0.199	(0.190–0.208)	0.953
**past 12 months**	40	(19.1–63.9)	–	–	0.384	(0.381–.0386)	31	(15.3–50.8)	–	–	0.291	(0.283–0.299)	-^d^
Methadone/men													
**past 72 h**	21.1	(6.05–45.6)	100	(99.2–100)	0.210	(0.199–0.208)	24	(9.36–45.1)	100	(98.2–100)	0.220	(0.202–0.237)	0.817
**past 12 months**	42.1	(20.3–66.5)			0.402	(0.399–0.405)	36	(18–57.5)	–	–	0.327706	(0.312–0.343)	-^d^
Methadone/women													
**past 72 h**	0.000	-^b^	100	–	0.00	–	0.00	–	100	–	0.00	–	–
**past 12 months**	0.000	-^b^	–	–	0.00	–	0.00	–	–	–	0.00	–	–
amphetamine and Methamphetamine/men													
**past 72 h**	0.000	-^b^	100	–	0.00	–	0.00	–	100	–	0.00	–	–
**past 12 months**	0.000	-^b^	–	–	0.00	–	0.00	–	–	–	0.00	–	–

^a^ Specificity was not calculated for past-12-month use because of the potential for inflated false negatives

^b^ Confidence interval could not be calculated because sensitivity was zero

^c^Chi-Square test: Comparison of self-reported substance use in two settings among urinalysis-confirmed users

^d^*p*-value was not calculated to compare of self-reported substance use over the past year, as some people may have correctly self-report of substance use over the past year, but a urine test could not determine substance use over the past year

Although, overall, the validity of self-reported opium and methadone use in men who were in the PHC group was lower than men in the RYCS group, but there was no statistically significant difference between the two groups in this regard.

For example, the use of opium and its derivatives in the RYCS group in comparison with the results of urine test, only about 37.5% of men with a positive test results had a true self-report substance use over the past 72 h. In the PHC group, about 53.3% of men over the 72 h had a true self-report substance use; However, the results of chi-square test showed that there was no statistically significant difference between the two groups in this regard (*p* = 0.211).

In addition, in the last 12 months, these ratios have reached about 56.3 and 70% for RYCS and PHC groups, respectively.

For substance other than opium, the sensitivity of self-reporting was lower but increased slightly with longer recall periods in both groups. The sensitivity of self-reporting about opium use over the past 72 h for women in RYCS group and PHC group were 100 and 25%, respectively. In general, underreporting for opium use was higher in women in the PHC group than in the RYCS group. However, it should be noted that while interpreting these results, among women who had a positive urine test (1 woman in RCYS group and 4 woman in PHC group), only a small minority of women (only 1in each group) had a true self-report opium use during the previous 72 h. This may explain non-concordance between two settings. However, the results showed that there was no statistically significant difference between the two groups in this regard (*p* = 0.171).

The specificity of self-reporting about substance use (i.e., number of negative self-reported divided by those with negative urine tests) for all drugs in both gender and both groups were > 99%. Conditional kappa or the level of agreement between self-report substance use over the past 72 h and urine tests revealed fair agreement for opium and methadone and slight for cannabis in men who were in the RYCS group and indicated moderate, fair and slight agreement for opium, methadone and cannabis in men in the PHC group, respectively.

For all substances, conditional kappa increased with longer recall periods. Also, in women, level of agreement between Self-reported substance use over the past 72 h and urine test indicated almost perfect agreement and fair for opium in RYCS and PHC groups, respectively.

Notably, in both groups who had given a urine sample, there were no reports of amphetamine and methamphetamine in both genders as well as for methadone and cannabis in women. While, prevalence estimated using urine test for methadone use in women were 0.88% (only one woman) and 2.34% (four women) in RYCS and PHC groups, respectively and for amphetamine and methamphetamine use in men were 0.20% (only one man) and 0.87% (two men) in RYCS and PHC groups, respectively. (Also, Supplementary Table 1, Additional file, shows concordance value between self-report substance use in different recall periods and having a positive urine test after combine both groups.)

Finally, Table 4 presents results of the multivariable logistic regression model for self-reporting substance use in group of PHC compared to group of RYCS. Although, relative to group of RYCS, group of PHC had greater odds of self-reported substance use during the past 72 h, but was no significant difference between the two groups in this regard.

**Table 4:** Multivariable logistic regression model of self-reporting substance use in during the past 72 h among urinalysis-confirmed users

	AOR^a^	95% CI	p
**Self-reported drug use during the past 72 h**			
PHC group (Ref = RYCS group)	1.25	(0.56–2.77)	0.590

^a^adjusted for gender (male/ female)

dds ratio was not calculated to compare of self-reported substance use over the past year, as some people may have correctly self-report of substance use over the past year, but a urine test could not determine substance use over the past year

## Discussion

In the present study, the effect of data collection settings, research setting versus primary health care setting, on the estimated prevalence of substance use and reporting bias in the two groups of youth and adolescents were investigated. From this perspective, the best of our knowledge, the current paper is the first study in the youth and adolescent population. It should be noted that sample in this study was selected from a geographical area with similar socioeconomic and cultural characteristics. Self-reported opium and methadone use over the past 72 h in men who were in the PHC group was higher than men in RYCS (research setting) group. Also the prevalence estimates based on urine test for all substances were higher in men who were in PHC group. As well as, there was a significant difference between the two groups of women in favor of opium use over the past 12 months. In other words, women who were in the RYCS group had lower self-report of opium use over the past 12 months.

The level of agreement between self-reported substance use over the past 72 h and urine test indicated fair and moderate agreement for opium in research and primary health care settings in men, respectively. In addition, the level of agreement between self-reported substance use over the past 72 h and urine test were fair for methadone and slight for cannabis in both settings. Conditional kappa indicated that in research setting versus primary health care setting, men were less willing to self-reported opium use.

Clark and colleagues demonstrated that individuals are usually more honest about substance use when the target is treatment. However, individuals tend to deny substance use when the target is not treatment [27].

In women, the results were the opposite, in which level of agreement between self-reported substance use over the past 72 h and urine test indicated almost perfect and fair agreement for opium in research setting and primary health care setting, respectively. Also, the level of agreement for methadone in both research setting and primary health care setting were slight. Nevertheless, overall, based on result of multivariable logistic regression model about self-reporting of substance use in over the past 72 h among urinalysis-confirmed users, although, relative to group of RYCS, group of primary health care (PHC) had greater odds of self-reported substance use, but was no significant difference between the two groups in this regard.

Although the answer to any sensitive question is expected to be influenced by the data collection settings, but it is more influenced by factors such as desirability and social stigma, public opinion about substance use, or potential legal sanctions, and may lead to reluctance for participants to report use [28]. Behaviors that are illegal, stigmatized, or have moral consequences may be less likely to be reported.

One way to evaluate whether self-reports substance use are affected by social desirability or legal sanction is to compare reports obtained under different methods that have various levels of privacy and anonymity. In various methodological studies, the higher self-reported substance use was observed in a study that provided the highest level of privacy. In general, prevalence estimates increase as privacy and confidentiality increase [29]. In this study, similar method and similar levels of privacy and anonymity were used to evaluate substance use in the two different settings (research and non-research) that indicated there was no significant difference between the two settings in respect the validity of self reported substance use, especially regarding certain substances, which are more stigmatized.

The results of this study led to concerning conclusions. We observed low sensitivity and high specificity of self-reported substance use compared to urine tests as the reference standard in the two settings, especially regarding certain substances, which are more stigmatized. Our results confirmed the general belief that people who are not drug users often tell the truth showing high specificity while some substance users who deny the real experience which lead to prove low sensitivity of self-reported substance use. These results were in line with by Ashrafi’s study. They showed that validity of self-reported substance use was low in Azar cohort as a population based study [4].

However, the validity of self-reported substance use over the past 72 h was lower in our study compared to other studies, which reported a good validity. Study by Abnet et al. reported a high sensitivity of opium use in Turkmen population in northern Iran [18]. Also, Zaldívar et al. showed a high validity of the self-report cannabis use and moderate validity for cocaine use in comparison with the urine test in college students [5]. Sensitivity and specificity are not constant and may vary for different setting, genders, age groups, samples and geographical areas as well as for different types of substances. Most possible explanation for this issue could be the beliefs and social and cultural differences for various regions and individuals [4, 27]. The amount and pattern of substance use denial vary for different substances, but generally, more stigmatized substances are denied more than less stigmatized substances.

This can be explained by the fact that respondents may be inconvenient to discuss about their substance use due to some reasons including public opinion, disgrace, embarrassment and fear of legal consequences [2, 22, 30]. Bradburn and Sudman have also reported the sensitive nature of substance use in a research conducted in the US, which indicated the inconvenience feeling to discuss about these topics, by respondents [31]. .Since consumed opium in this population was with less social stigma than other substances the validity of reporting of this substance was higher in compare to other substances which this finding are supported by some research results that indicating substance use underreporting increases with the level of its stigma [2, 22].

Finally, further analysis showed that validity of self-report substance use over the past year was higher compared to over the past 72 h in the two groups. However, it should be noted, that the urine test only detects substance use over the past 72 h and is not able to detect substance over longer periods. Therefore, it can be concluded that in people who have been using substance recently (urinalysis-confirmed users), questions regarding past substance use are likely to generate less underreporting in comparison with those concerning recent substance use. In a study, Harrison found that underreporting had little or no effect on self-reported lifetime use, more on self-reported use past year, and most on self-reported use last month. Consistent with the results of our study, Harrison Found that Valid self-reporting of substance use is a function of ‘1) the recency of the event, 2) the social desirability of the substance [32]. Thus, we suggest that the estimates of substance use in past year in self-report studies are likely more valid then past 72 h.

Some studies found that questions regarding past substance use are likely to generate less underreporting when compared with those concerning recent substance use [22, 33]. The contrast between recent and previous analysis indicated a general unwillingness to the recent self-report of substance use. Besides, these results support the concept that self-report substance use over the past year is more reliable than the recent use in the two settings (research and non-research).

Compared to biological markers, self-reported data is usually cheaper and more practical, allowing researchers to obtain and collect more information on long-term substance use, methods of use, and user behaviors. Also, relying on self-reported data is often the only appropriate approach to assess substance use, but responses may be influenced by factors such as report bias, social desirability, stigma and participant’s ability to recall information. Also, social disgrace associated with substance use or potential legal sanction may lead participants to be reluctant to report the use of these illicit substances, or individuals may even exaggerate experience of substance use because of gaining certain privileges in some conditions [4, 34]. Therefore, for such population, according to our results, in two setting, research and primary health care, underreporting cannot be overlooked and studies on sensitive issues such as substance use should be taken into careful consideration in respect of false negative, thus this bias needs to be estimated and corrected. Otherwise it could lead to finding a spurious association.

### Limitations

The main limitations of the present study were listed as following:

- In order to influence the data collection environment on the validity of self-report substance use, individuals were not randomly assigned to the groups. Therefore, other factors may also play a role in estimating substance use in both environments. For example, participants in the PHC group were those who had declined to participate the RYCS, thus some difference may be due to personality differences between the two groups, although they were selected from a geographical area with similar socioeconomic and cultural characteristics.
- Appropriate markers are available to identify drug metabolites for longer periods of time. However, urine test is considered as a simple, fast, and inexpensive method for assessing the validity of self-reported substance use in the epidemiological studies. There were no protocols for the use of other biological samples such as hair at the time of this study.
- People who have been passively exposed to drugs were not investigated.

## Conclusion

Individuals in primary health care setting were more likely to self-reported substance use than in research setting, but setting did not have a statistically significant effect in terms of self-reported substance use. Programs that rely on self-reported substance use may not estimate the exact prevalence of substance use in both research and primary health care settings, especially substances that have a higher social stigma. Therefore, it is recommended that self-report and biological indicators be used for more accurate evaluation in substance use studies. It is also suggested that future epidemiological studies be performed to reduce bias of social desirability and find a method providing the highest level of privacy.

## Supplementary Information

**Additional file 1: Supplementary Table 1.** Concordance between self-reported use of substance and urine test at among participants, combine both groups (*N* = 1007).

## Data Availability

The datasets used during the current study are available on the RafsanjanYouth Cohort Study Center, Rafsanjan University of Medical Sciences, Iran. The data is not available publicly. However, upon a reasonable request, the data can be obtained from the authors.

## Abbreviations

RYCS: Rafsanjan youth cohort study
PHC: Primary health care
CI: Confidence interval
