# Supplementary material for: Validity of self-reported substance use: research setting versus primary health care setting
Source: Subst Abuse Treat Prev Policy. 2021 Sep 14;16:66. doi: 10.1186/s13011-021-00398-3 (PMC8439053; doi:10.1186/s13011-021-00398-3)
Supplement: Supplementary file 1 — Additional file 1: Supplementary Table 1. Concordance between self-reported use of substance and urine test at among participants, combine both groups (N = 1007). [file 13011_2021_398_MOESM1_ESM.docx]

| **Supplementary Table 1: Concordance between self-reported use of substance and urine test at among participants, combine both groups (N = 1007).** | | | | | | |
| --- | --- | --- | --- | --- | --- | --- |
| Drug/gender | Sensitivity | 95% CI | Specificity^a^ | 95% CI | Conditional κ | 95% CI |
| Opioid/total | | | | | | |
| **past 72 hours** | 44.8% | (32.6% - 57.4%) | 100% | (99.6% -100%) | 0.431 | (0.427-0.435) |
| **past 12 months** | 61.2% | (48.5% - 72.9%) | - | - | 0.569 | 0.566-0.571)) |
| Opioid/men | | | | | | |
| **past 72 hours** | 45.2% | (32.5%- 58.3%) | 100% | (99.4% -100%) | 0.430 | 0.423-0.436)) |
| **past 12 months** | 62.9% | (49.7% -74.8%) | - | - | 0.574 | 0.571-0.578)) |
| Opioid/women | | | | | | |
| **past 72 hours** | 40% | (5.27% -85.3%) | 100% | (98.7% -100%) | 0.400 | (0.394-0.398) |
| **past 12 months** | 40% | 5.27% 85.3% |  |  | 0.382609 | 0.382-0.384)) |
| Cannabis/ men | | | | | | |
| **past 72 hours** | 11.5% | (2.45% - 30.2%) | 100% | (99.5% -100%) | 0.112 | 0.108-0.115)) |
| **past 12 months** | 15.4% | (4.36% - 34.9%) | - | - | 0.141916 | 0.140-0.144)) |
| Methadone/total | | | | | | |
| **past 72 hours** | 20.4% | (10.2% - 34.3%) | 100% | (99.6% -100%) | 0.196 | 0.192-0.200)) |
| **past 12 months** | 34.7% | (21.7% - 49.6%) | - | - | 0.329 | 0.326-0.332)) |
| Methadone/men | | | | | | |
| **past 72 hours** | 22.7% | (11.5% -37.8%) | 100% | (99.5% -100%) | 0.216 | (0.211-0.222) |
| **past 12 months** | 38.6% | (24.4%- 54.5%) | - | - | 0.363 | 0.358-0.367)) |
| Methadone/women | | | | | | |
| **past 72 hours** | 0.000 | -^b^ | 100 | - | 0.000 | - |
| **past 12 months** | 0.000 | -^b^ | - | - | 0.000 | - |
| amphetamine and Methamphetamine/men | | | | | | |
| **past 72 hours** | 0.000 | -^b^ | 100 | - | 0.00 | - |
| **past 12 months** | 0.000 | -^b^ | - | - | 0.00 | - |
| ^a^ Specificity was not calculated for past-12-month use because of the potential for inflated false negatives.  ^b^ Confidence interval could not be calculated because sensitivity was zero. | | | | | | |
